# Content of free and protein‐binding N^ε^‐carboxymethyllysine and N^ε^‐carboxyethyllysine in different parts of braised chicken

**DOI:** 10.1002/fsn3.1317

**Published:** 2020-01-09

**Authors:** Zongshuai Zhu, Rui Fang, Yiqun Cheng, Iftikhar Ali Khan, Jichao Huang, Bin Li, Ming Huang

**Affiliations:** ^1^ Nanjing Innovation Center of Meat Products Processing Jiangsu Collaborative Innovation Center of Meat Production and Processing, Quality and Safety Control College of Food Science and Technology Nanjing Agricultural University Nanjing China; ^2^ College of Engineering Nanjing Agricultural University Nanjing China; ^3^ Science and Technology Cooperation Center Jiyuan China

**Keywords:** braised chicken, correlation, N^ε^‐carboxyethyllysine, N^ε^‐carboxymethyllysine, oxidation

## Abstract

In order to illustrate the levels of advanced glycation end products (AGEs) in Chinese traditional braised chicken**,** the distribution of free and protein‐binding N^ε^‐carboxymethyllysine (CML) and N^ε^‐carboxyethyllysine (CEL) in four parts of processed chicken including chest (X), leg (T), skin (P), and the mixed whole body (M) was investigated. Our results showed that the content of free CML was 1,186.63–1,795.43 ng/g meat and protein‐binding CML was 11,693.91–16,122.90 ng/g meat. Differently, the content of free CEL was 24.81–41.62 ng/g meat and protein‐binding CEL was 270.11–385.49 ng/g meat. It was found that the total contents of CML were 31.5–56.8 folds higher than those of CEL. Protein‐binding AGEs (CML + CEL) were 6.6–9.9 times higher than those of free AGEs (CML + CEL). Pearson's correlation of AGEs and oxidation in four parts of braised chicken were also investigated, and the results showed that oxidation had a significant effect on levels of CEL; especially, the protein carbonyl was negatively correlated with free CEL (*p* < .05). TBARs value was significantly positively correlated with protein‐binding and total CEL (*p* < .01). In conclusion, our findings are important for better understanding of the AGEs formation in braised meat.

## Graphical abstract

CML and CEL in four parts of braised chicken including chest (X), leg (T), skin (P) and the mixed whole (M) were measured. We found that the total content of CML were 31.5–56.8 folds higher than that of CEL. Protein‐binding AGEs (CML + CEL) were 6.6–9.9 times higher than that of free.

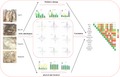

## 1 INTRODUCTION

Braised chicken is a representative of traditional Chinese meat products, of which delicious taste is formed by typical thermal processing including deep‐frying (160–180°C) and water boiling (90–95°C). Braised chicken has been an indispensable dish for Chinese consumers (Duan et al., 2015; Liu et al., 2017). However, the Maillard reaction is extremely promoted resulting in a large number of harmful substances due to the longtime thermal processing of braised chicken. Advanced glycation end products (AGEs) are one of the series of harmful substances for human health, which are largely formed at the advanced stage of the Maillard reaction (Poulsen et al., 2013; Sebeková & Somoza, 2007). N^ε^‐carboxymethyllysine (CML) and N^ε^‐carboxyethyllysine (CEL) are the two main components of AGEs, because they have been studied most clearly, and they are found in high levels in meat products (Glj, Woodside, Ames, & Cuskelly, 2012; Sun et al., 2015; Uribarri et al., 2010). According to the AGEs forms in food, CML and CEL can be divided into two categories: One is the free form existing on the surface of meat and with separation of the precursor substance; another is the protein‐binding form, which is covalently bonded with proteins and peptides (Niu et al., 2018; Sun et al., 2016). The digestion and absorption effects of food‐derived free and protein‐binding AGEs are different. Therefore, it is necessary to separate them and study the content distribution and formation in detail (Ahmed et al., 2010; Naila & Thornalley, 2012). However, few publications reported the content and distribution of free and protein‐binding forms of CML or CEL in Chinese traditional processed meat products, especially on braised chicken.

There are many factors influencing the formation of CML and CEL; two common factors are thermal processing conditions and the levels of precursor substances such as protein, fat, and water (Sun et al., 2015). Processing temperature has a significant effect on the formation of AGEs. Chen & Smith compared beef, pork, chicken, and fish CML generation under frying (204℃), broiling (232℃), and baking (177℃) conditions (Chen & Smith, 2015). The results showed that higher temperatures produce higher CML. In addition, CML and CEL can be generated in large quantities not only through the Maillard reaction, but also through the oxidation pathway such as lipid oxidation (Poulsen et al., 2013). High processing temperature not only promotes the oxidation of fat in meat products but also generates a large number of reactive oxygen radicals, which will further promote the Maillard reaction to produce active α‐dicarbonyl compounds, such as methylglyoxal (MGO) and glyoxal (GO) (Degen, Hellwig, & Henle, 2012; Jiang, Hengel, Pan, Seiber, & Shibamoto, 2013; Sheng, Larsen, Le, & Zhao, 2018). However, most published works have only studied the AGEs of protein‐binding in meat or the mixture of free and protein‐binding forms; few studies divided the AGEs of free and protein‐binding forms separately. (Gong, Guangwei, Lu, & Mitchell, 2011). Meanwhile, very few publications reported the levels and distribution of free and protein‐binding forms of AGEs in processed Chinese traditional meat products. Thus, the formation mechanism and content distribution of free and protein‐binding forms of CML and CEL in heat‐treated meat are still dim, especially on braised chicken. So, the purpose of this study is to determine the content of free and protein‐binding forms of AGEs, mainly including CML and CEL in braised chicken breast (X), leg (T), skin (P), and the whole body (M). Furthermore, the correlation of protein and fat oxidation on different forms of AGEs in braised chickens were also studied. These findings provided references for people to select low content of AGEs in braised chicken products.

## 2 MATERIALS AND METHODS

### 2.1 Materials and reagents

Five famous brands of braised chicken named H (purchased from Nanjing, Jiangsu in China), L (purchased from Suzhou, Anhui in China), K (purchased from Daokou, Henan in China), D (purchased from Dezhou, Shandong in China), and N (purchased from Nanjing, Jiangsu in China) were used as measure samples. 5,5′‐Dithiobis (2‐nitrobenzoic acid), 99% DTNB, 2,4‐dinitrophenylhydrazine (DNPH), trichloroacetic acid (TCA), thiobarbituric acid (TBA), guanidine hydrochloride, hydrochloric acid, sodium hydroxide, ethyl acetate, ethanol, methanol, ammonium hydroxide, 1,1,3,3‐Tetrathoxypropane, sodium tetraborate decahydrate, sodium borohydride, ethylenediaminetetraacetic acid disodium salt (EDTA‐Na_2_), phosphate buffer saline (PBS, pH7.2–7.4), n‐hexane, and BCA kit were purchased from Nanjing Ruiyi Biological Technology Co., Ltd. The solid phase extraction (SPE) columns (Oasis MCX cartridge, 60 mg/3 ml, 30 μm) were obtained from Waters Corporation. Chicken ELISA kit was purchased from Nanjing Maibo Biotechnology Co., Ltd, China.

### 2.2 Composition analyses

The four parts including X, T, P, and M of the five brands of braised chicken were cut into 1 cm^3^ piece of meat. Then, they were put into the homogenate machine (Ultra Turrax T25 BASIS, German IKA Company) for crushing. Crude protein was determined by Kjeltec™^2300^ (Denmark FOSS company) according to AOAC Int method 992.15 (King‐Brink & Sebranek, 1993). Fat content and moisture content were determined by AOAC Int method 2008.06 (Leffler et al., 2008).

### 2.3 Sample preparation for analysis of free CML and CEL

1‐g meat (with an error of 0.001 g) was accurately weighed and put into 50‐ml centrifuge tube. The free CML and CEL were determined by Sun and Niu's method with some modifications (Niu et al., 2018; Sun et al., 2015). Precooled 5% TCA was added into the centrifugal tube and mixed with glass rods. A homogenate continuously at 2000g for 2 times was taken under the condition of ice bath; each homogenate time lasted 30 s. Then, the sample was centrifuged at 1000g for 10 min and the supernatant was added with 15‐ml n‐hexane; it was vibrated several times to be mixed before centrifugation at 5,000 rpm for 15 min. The upper fat was discarded, and the lower layer was collected into a 10‐ml polyethylene (PE) pipe. Finally, the sample was purified by MCX column.

### 2.4 Sample preparation for analysis of protein‐binding CML and CEL

0.2‐g meat (with an error of 0.001 g) was accurately weighed and put into a 50‐ml centrifuge tube. The protein‐binding CML and CEL were determined by Sun's method with some modifications (Sun et al., 2015). Samples were put under the condition of 4℃ by adding sodium borate buffer solution and sodium borohydride solution to reduction for a night. Then, 20% TCA and n‐hexane were added into the tube and blended before 10,000 *g*, 30 min centrifugation, and then, the supernatant was discarded and repeated 3 times. The sample was transferred to a pressure bottle with 50℃ nitrogen blow to dry. 6 M hydrochloric acid was added into a pressure bottle for sample acid hydrolysis about 24 hr, then the sample was placed in the oven to 110℃ using nitrogen sealed. Finally, the hydrolysate was filtered, and dried at 50℃ using nitrogen, then the dried sample was redissolution with water and purified by MCX column.

### 2.5 Chicken ELISA analysis

Chicken ELISA kit was used to determine the free and protein‐binding states of CML and CEL samples after purified by MCX column. The measurement method was slightly modified according to the kit instructions. Samples with high concentration were diluted to fall within the linear range of the standard curve (10–320 ng/ml CML, *R* ^2^ = .9994; 0.25–8 ng/ml CEL, *R* ^2^ = .9998). In addition to the blank hole, standard and sample holes were set up. Each hole was added with 100‐μl horseradish peroxidase (HRP)‐labeled detection antibody, under 37℃ dark reaction for 60 min. At the end of the reaction, the liquid was discarded and 350‐μl washing solution was added into each hole. It was kept for 1 min, and the liquid was forcefully discarded. Finally, the above steps were repeated 5 times. Then, 50‐μl A and B reaction liquid substrates were added into each hole keeping 37℃ in dark conditions for 15 min. In the end, 50‐μl terminated liquid was added immediately and the absorbance was determined by microplate reader at 450 nm of each hole.

### 2.6 TBARs measurement

TBARs value was measured according to the method of Uchiyama and Yu et al., with slight modifications (Uchiyama & Mihara, 1978; Yu, He, Zeng, Zheng, & Chen, 2016). 4 g of meat samples from X, T, P, and M parts was accurately weighed and taken into a 50‐ml centrifuge tube, respectively. 20 ml of 7.5% TCA contained EDTA‐Na_2_ was added to the tube. 10,000 rpm homogenate was taken for mixing each other under ice bath condition. The protein concentration of protein extract was determined by BCA kit. MDA standard curves of different concentrations were prepared using 1,1,3,3‐Tetrathoxypropane, TBA (0.02 mol), and TCA (7.5%) contained EDTA‐Na_2_. 2 ml of protein extraction and 2 ml of TBA were added into a 10‐ml PE tube with reaction for 30 min under 95℃ water bath. After water cooled to room temperature, samples were measured by using microplate reader. The results were expressed as mg MDA/kg meat.

### 2.7 Carbonyl content

Applied method was described by G. Liu, Xiong, & Butterfield with slight modification (Liu, Xiong, & Butterfield, 2010). Meat samples from different parts of X, T, P, and M were dissolved in PBS (0.01 mol, pH7.4), and the protein concentration was determined by BCA kit. 1‐ml of protein solution was sucked up into a 10‐ml PE tube, and 10 mmol/L DNPH solution (hydrochloric acid concentration is 2 mol/L) was added. After 1‐hr reaction at room temperature away from light, 2 ml of 20% TCA was added and centrifuged for 5 min (10,000 *g*). Supernatant was discarded, and 5 ml of ethyl acetate/ethanol (v:v = 1:1) was also precipitated to wash three times; then, the sample was dissolved by the 6 mol/L guanidine hydrochloride and put at room temperature for 30 min as well as centrifuged for 10 min (10,000 *g*). The supernatant was taken, the absorbance at 370 nm was determined by microplate reader, and carbonyl was expressed as nmol/mg protein.

### 2.8 Active sulfhydryl

Xue's method to determine the active sulfhydryl group was slightly modified (Xue et al., 2017). Meat samples from different parts of X, T, P, and M were dissolved in PBS (0.01 mol, pH7.4), and the protein concentration was determined by BCA kit. 0.4‐ml protein sample was added to 4.6‐ml PBS and 20‐μl DTNB, which was shaken evenly, and incubated at room temperature for 1 hr, and the absorbance was determined at 412 nm with microplate reader. Active sulfhydryl content was expressed as μmol/mg protein.

### 2.9 Statistical analysis

All experiments were determined with three repetitions (*n* = 3) and expressed as mean ± *SD*. SAS analysis software (SAS software research institute, USA, version 8.1) was used for statistical analysis of data, one‐way ANOVA method was used for analysis of variance, and Duncan's Multiple Range test was used to compare the differences between mean values. *p* < .05 indicates a significant difference in the results. Pearson's correlation was used by SPSS analysis software (Version 16; IBM Corp. Armonk, NY) to investigate the relationship between oxidation and the different forms of AGEs.

## 3 RESULTS AND DISCUSSION

### 3.1 Samples composition and oxidation

Detailed results of protein content, fat content, water content, carbonyl group, active sulfhydryl, and TBARs in five braised chickens X, T, P, and M are shown in Table 1. The average protein content of X, T, P, and M of the five braised chicken was 14.27%–28.68%, among which T had the highest protein content and P had the lowest. The average content of fat was between 6.85% and 43.47%, among which P was significantly different from X, T, and M (*p* < .05), and fat content of P was the highest. The fat content of T was higher than that of X, but there was no significant difference (*p* > .05). The average moisture content of X was the highest but not significantly different from P and M, while T was significantly different from X, P, and M (*p* < .05). The oxidative indexes of carbonyl, active sulfhydryl, and TBARs were not significantly different in X, T, P, and M. In addition, the TBARs value of P was the highest, and T was more than X.

**Table 1:** Average content of protein, fat, moisture, carbonyl, active sulfhydryl, and TBARs from five brands of braised chicken, *n* = 3, mean ± *SD*

Brand	Part	Protein (%)	Fat (%)	Moisture (%)	Carbonyl (nmol/mg protein)	Active sulfhydryl (μmol/mg protein)	TBARs (mg MDA/kg meat)
H	X	23.8213 ± 1.5603^b^	13.7783 ± 3.6731^c^	63.2927 ± 0.6295^a^	4.0124 ± 0.0153^a^	0.0245 ± 0.0006^a^	0.0811 ± 0.0068^b^
	T	28.5801 ± 3.9354^a^	17.4668 ± 0.2595^b^	62.8088 ± 0.0894^a^	1.5953 ± 0.4130^b^	0.0148 ± 0.0021^b^	0.1100 ± 0.0085^a^
	P	7.5244 ± 1.0860^c^	48.9914 ± 2.6823^a^	50.3325 ± 0.3464^c^	3.3787 ± 0.4164^a^	0.0267 ± 0.0052^a^	0.1216 ± 0.0107^a^
	M	26.2869 ± 1.0136^b^	12.2280 ± 0.8707^c^	60.6504 ± 1.0754^b^	2.8825 ± 0.6506^a^	0.0152 ± 0.0011^b^	0.1117 ± 0.0075^a^
N	X	23.7861 ± 2.1970^a^	4.0154 ± 0.4337^d^	61.6604 ± 0.0180^a^	1.8256 ± 0.3095^a^	0.0284 ± 0.0008^a^	0.2677 ± 0.0093^b^
	T	25.8005 ± 2.3774^a^	4.6812 ± 0.3129^c^	61.4754 ± 0.1311^a^	1.4543 ± 0.3186^ab^	0.0196 ± 0.0012^c^	0.3532 ± 0.0221^a^
	P	9.7642 ± 0.8362^b^	43.6274 ± 2.8466^a^	51.2121 ± 0.4801^c^	1.8646 ± 0.2290^a^	0.0172 ± 0.0021^d^	0.1026 ± 0.0123^c^
	M	25.8792 ± 0.5221^a^	11.0857 ± 1.4327^b^	60.3072 ± 0.1434^b^	1.1303 ± 0.2745^b^	0.0225 ± 0.0007^b^	0.2619 ± 0.0162^b^
D	X	31.8491 ± 1.7967^a^	4.4988 ± 1.7047^d^	64.3473 ± 0.0691^a^	2.2779 ± 0.4577	0.0253 ± 0.0016^a^	0.0676 ± 0.0027^c^
	T	30.7384 ± 5.5736^ab^	13.1991 ± 0.8760^c^	62.8364 ± 0.0372^b^	1.9246 ± 0.2680	0.0241 ± 0.0048^a^	0.0915 ± 0.0154^b^
	P	21.6618 ± 1.7861^b^	38.5998 ± 2.9390^a^	50.7158 ± 0.6355^d^	2.0812 ± 0.5332	0.0182 ± 0.0024^b^	0.1697 ± 0.0037^a^
	M	23.6761 ± 0.5218^b^	16.9231 ± 0.7165^b^	61.6144 ± 0.1042^c^	2.2618 ± 0.4682	0.0254 ± 0.0007^a^	0.1006 ± 0.0285^c^
K	X	30.2873 ± 0.5219^b^	3.8506 ± 0.7290^d^	62.4729 ± 0.0609^a^	3.7396 ± 0.1339^a^	0.0225 ± 0.0037^c^	0.0582 ± 0.0005^d^
	T	28.9837 ± 3.0500^c^	9.8122 ± 2.5133^c^	60.3477 ± 0.0468^b^	3.1939 ± 0.2553^a^	0.0297 ± 0.0097^bc^	0.0619 ± 0.0042^c^
	P	14.3244 ± 0.8564^d^	48.8614 ± 1.3278^a^	48.2857 ± 1.4661^d^	1.6998 ± 0.0614^b^	0.0263 ± 0.0027^b^	0.1451 ± 0.0142^a^
	M	32.1952 ± 1.4150^a^	16.8544 ± 0.9460^b^	58.5603 ± 0.0323^c^	3.3491 ± 0.5175^a^	0.0392 ± 0.0074^a^	0.0755 ± 0.0090^b^
L	X	30.1955 ± 0.5226^a^	8.1278 ± 0.8673^c^	60.0906 ± 0.2317^b^	5.4782 ± 0.8111^a^	0.0509 ± 0.0022^b^	0.0572 ± 0.0022^c^
	T	29.3343 ± 3.3270^b^	8.4176 ± 1.0076^c^	58.9491 ± 0.1628^c^	3.0979 ± 0.5789^c^	0.0340 ± 0.0067^c^	0.0583 ± 0.0046^c^
	P	18.1187 ± 1.5671^c^	37.2896 ± 2.2600^a^	61.4316 ± 0.3947^a^	3.9068 ± 0.2533^bc^	0.0553 ± 0.0100^a^	0.2021 ± 0.0358^a^
	M	31.9439 ± 2.3095^a^	12.6012 ± 1.5737^b^	59.8517 ± 0.0718^b^	4.4391 ± 0.2738^ab^	0.0389 ± 0.0007^c^	0.0923 ± 0.0045^b^

Note

Comparison of different parts of the same brand with the same index, marked a, b, c, and d with different letters, indicates significant difference (*p* < .05).

According to the results, X and T in braised chicken mainly consisted of protein and P was mainly fat, and the fat oxidation degree of T was greater than that of X, which meant that protein oxidation and fat oxidation mainly occurred in different chicken parts due to the thermal processing. This result was consistent with Rashmi's report (Rashmi, Deepthi, & Modi, 2013).

### 3.2 Free and protein‐binding CML and CEL in braised chicken

**Figure 1:**
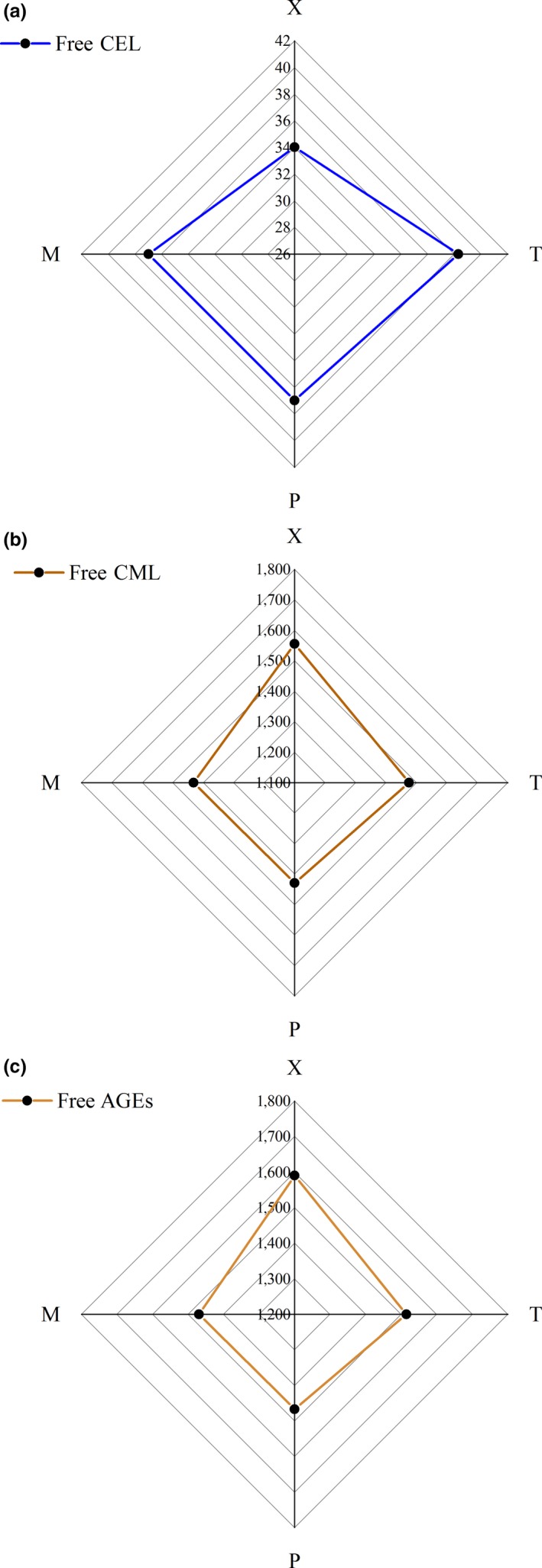
Radar map of breast (X), leg (T), skin (P), and the whole body (M) (four parts) with different existence forms of free CML, CEL, and AGEs (CML + CEL), *n* = 3, ng/g meat. (a) Free CEL; (b) Free CML; (c) Free AGEs

The distribution of free CML and CEL in different parts of braised chicken is shown in Figure 1. For the levels of free CML, X was the highest and the average value was 1,556.517 ± 142.542 ng/g meat; P was the lowest, with an average of 1,429.331 ± 41.392 ng/g meat. For the levels of free CEL, T was the highest, with an average of 38.284 ± 1.955 ng/g meat, and X was the lowest, with an average of 34.027 ± 5.885 ng/g meat. For the levels of free AGEs (CML + CEL), T had the highest value, and the mean value was 1,590.546 ± 142.334 ng/g meat. P had the lowest, with an average value of 1,466.309 ± 40.344 ng/g meat.

**Figure 2:**
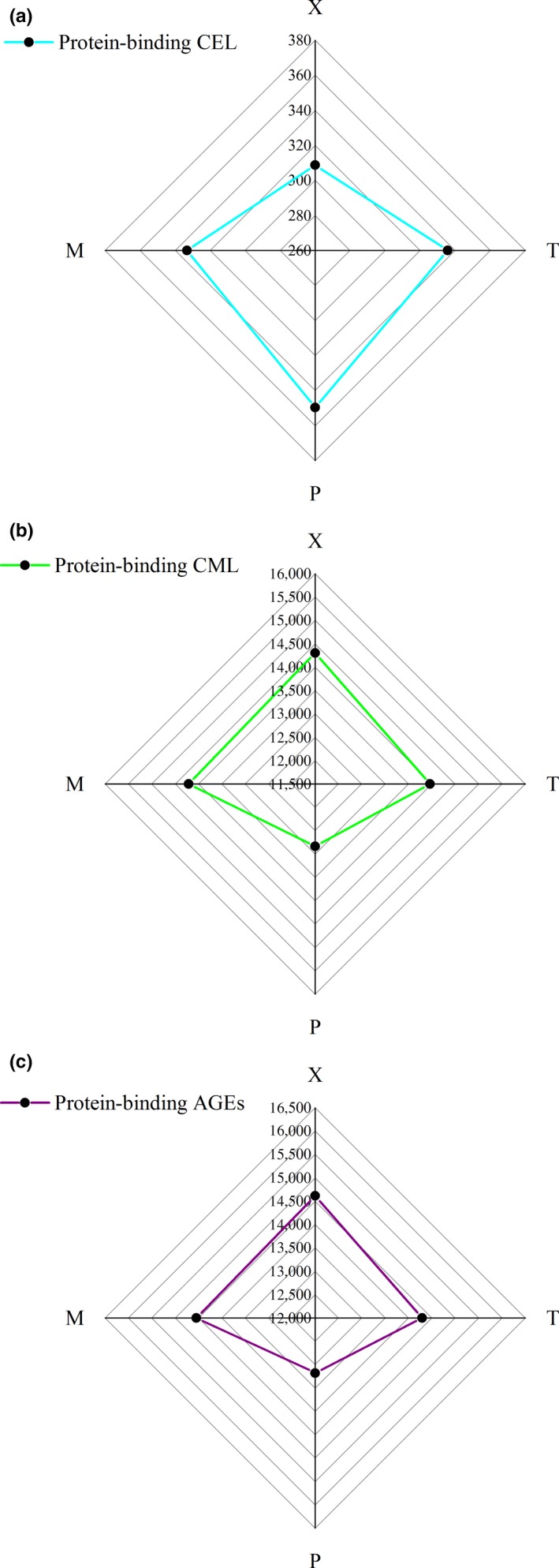
Radar map of breast (X), leg (T), skin (P), and the whole body (M) (four parts) with different existence forms of protein‐binding CML, CEL, and AGEs (CML + CEL), *n* = 3, ng/g meat. (a) Protein‐binding CEL; (b) Protein‐binding CML; (c) Protein‐binding AGEs

The distribution of protein‐binding CML and CEL in different parts of braised chicken is shown in Figure 2. X had the highest protein‐binding CML, with an average value of 14,310.240 ± 1,156.602 ng/g meat. P was the lowest, with an average value of 12,832.700 ± 741.523 ng/g meat, and P was a significant difference between X and T (*p* < .05). For levels of CEL, X was the lowest, with an average of 308.767 ± 24.443 ng/g meat, and there was a significant difference on X, T, and P (*p* < .05), but there was no significant difference between T and P. Moreover, P was the highest, with an average value of 335.644 ± 14.455 ng/g meat. Furthermore, P showed the lowest content of protein‐binding AGEs (CML + CEL), and the mean value was 13,182.350 ± 750.067 ng/g meat, which was significantly different from X, T, and M (*p* < .05). X was the highest, with an average value of 14,617.230 ± 1,164.637 ng/g meat, but there was no significant difference between X, T, and M (*p* > .05).

**Figure 3:**
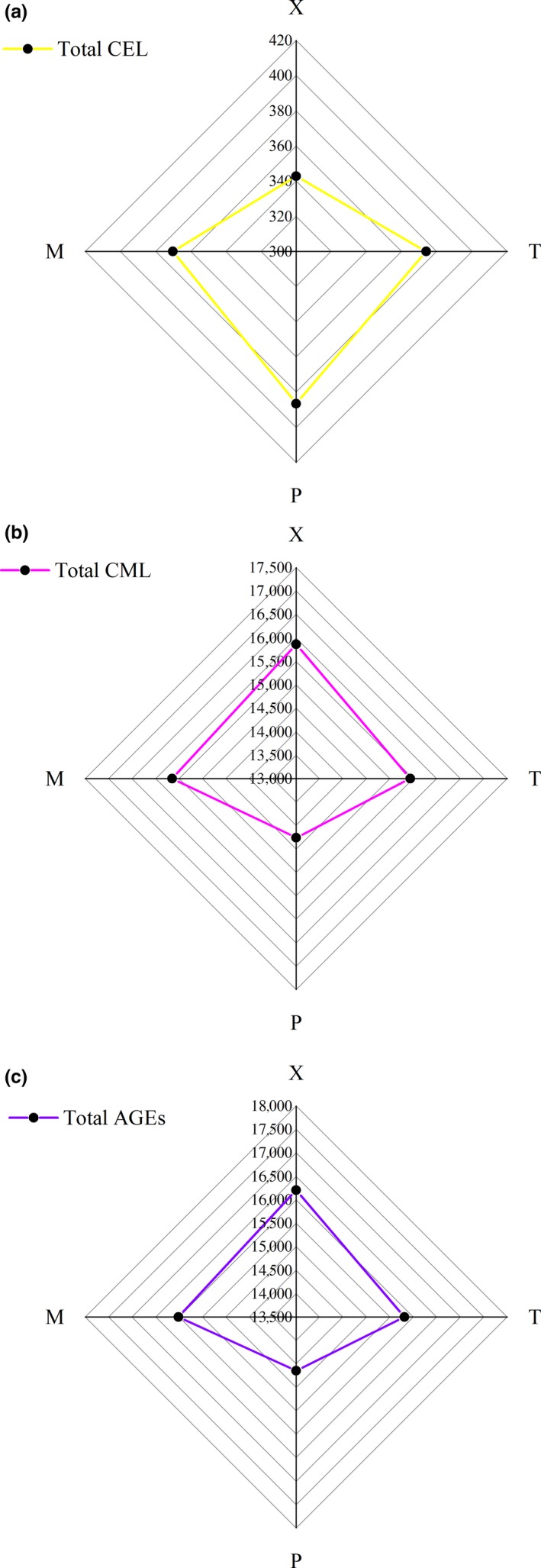
Radar map of breast (X), leg (T), skin (P), and the whole body (M) (four parts) with different existence forms of total CML, CEL, and AGEs (CML + CEL), *n* = 3, ng/g meat. (a) Total CEL; (b) Total CML; (c) Total AGEs

The distribution of total CML, CEL, and AGEs in different parts of braised chicken is shown in Figure 3. The total CML content in X was the highest, with an average value of 15,866.780 ± 1,067.230. P was the lowest, with an average value of 14,262.030 ± 734.3910 ng/g meat, and there was no difference between P and T. Compared with the mean values of total CEL, the content of X was the lowest, with an average value of 342.794 ± 12.56 ng/g meat. The content of P was the highest, with an average of 386.642 ± 18.297 ng/g meat. There was no significant difference between T, P, and M, but there was a significant difference between X and T, P, and M (*p* < .05). Compared with the means of total AGEs, for total AGEs content, P was the lowest, with an average of 14,648.670 ± 742.006 ng/g meat, and X was the highest, with an average of 16,209.570 ± 1,071.877 ng/g meat. Among X, T, and M, there was no significant difference, but P was significantly different from X, T, and M (*p* < .05). All the detailed results are shown in Table 2.

**Table 2:** Average content of free and protein‐binding CML, CEL, total CML, total CEL, free AGEs, protein‐binding AGEs, and total AGEs of five brands of braised chicken with four parts including chest (X), leg (T), skin (P), and the whole body (M). AGEs = CML + CEL. *n* = 3, mean ± *SD*

Brand	Part	CML (ng/g meat)		CEL (ng/g meat)		Total CML (ng/g meat)	Total CEL (ng/g meat)	Free AGEs (ng/g meat)	Protein‐binding AGEs (ng/g meat)	Total AGEs (ng/g meat)
		Free	Protein‐binding	Free	Protein‐binding	Free CML + protein‐binding‐binding CML	Free CEL + protein‐binding CEL	Free CML + freeCEL	Protein‐binding CML + CEL	Total CML + CEL
H	X	1,609.043 ± 96.293^b^	13,948.985 ± 10.626^b^	35.872 ± 0.032^c^	270.107 ± 0.292^c^	15,558.03 ± 99.182^b^	305.979 ± 0.259^c^	1,644.915 ± 96.322^b^	14,210.09 ± 10.456^b^	15,864.01 ± 98.934^c^
	T	1,469.87 ± 70.066^b^	14,175.07 ± 19.156^c^	40.573 ± 0.185^a^	330.516 ± 1.293^a^	15,644.94 ± 74.759^c^	371.090 ± 1.229^a^	1,510.443 ± 70.243^b^	14,505.59 ± 20.046^c^	16,016.03 ± 74.376^d^
	P	1,432.435 ± 46.6561^b^	12,497.39 ± 18.073^a^	41.619 ± 0.113^b^	346.916 ± 1.086^b^	13,929.83 ± 28.584^a^	388.535 ± 0.980^b^	1,474.054 ± 46.769^b^	12,844.31 ± 19.064^a^	14,318.366 ± 27.707^a^
	M	1,375.174 ± 2.884^a^	13,870.14 ± 86.631^b^	39.702 ± 0.005^d^	322.219 ± 0.254^d^	15,245.32 ± 85.551^a^	361.922 ± 0.259^d^	1,414.876 ± 2.879^a^	14,192.36 ± 86.836^b^	15,607.24 ± 85.755^b^
N	X	1,406.348 ± 81.237^b^	15,443.48 ± 30.915^c^	38.341 ± 0.048^c^	327.963 ± 0.590^d^	16,849.83 ± 13.158^c^	366.305 ± 0.584^c^	1,444.675 ± 3.260^b^	15,771.44 ± 30.330^c^	17,216.13 ± 12.804
	T	1,513.957 ± 2.213^b^	14,413.91 ± 35.300^d^	37.713 ± 0.083^d^	345.279 ± 0.667^a^	15,927.87 ± 19.430^d^	382.993 ± 0.696^a^	1,551.717 ± 2.240^b^	14,759.19 ± 35.580^d^	16,310.86 ± 20.398
	P	1,378.957 ± 2.213^a^	13,142.03 ± 35.528^b^	34.598 ± 0.070^b^	347.166 ± 1.396^b^	14,520.99 ± 9.592^b^	381.764 ± 1.404^a^	1,413.519 ± 2.167^a^	13,489.2 ± 36.474^b^	14,902.75 ± 11.1939
	M	1,363.435 ± 81.2372^b^	13,958.26 ± 68.425^a^	35.497 ± 0.028^a^	324.356 ± 0.508^c^	15,321.7 ± 142.253^a^	359.854 ± 0.523^b^	1,398.932 ± 81.259^ab^	14,282.62 ± 68.655^a^	15,681.55 ± 142.341
D	X	1,406.478 ± 31.667	14,514.78 ± 6.024^b^	31.888 ± 0.076^a^	299.854 ± 0.440^a^	15,921.26 ± 29.409^b^	331.743 ± 0.377^a^	1,438.376 ± 31.647	14,814.64 ± 6.457^b^	16,253 ± 29.334^b^
	T	1,564.696 ± 307.145	12,953.04 ± 21.721^d^	36.268 ± 0.086^b^	315.698 ± 0.554^b^	14,517.14 ± 286.146^c^	351.967 ± 0.468^b^	1,600.964 ± 307.188	13,268.74 ± 22.138^d^	14,869.71 ± 285.887^c^
	P	1,445.478 ± 33.549	11,693.91 ± 15.161^c^	38.138 ± 0.019^c^	334.540 ± 1.027^c^	13,139.39 ± 30.929^b^	372.678 ± 1.010^c^	1,483.617 ± 33.568	12,028.45 ± 16.111^c^	13,512.07 ± 30.619^b^
	M	1,440.522 ± 61.913	13,179.13 ± 13.913^a^	38.903 ± 0.052^d^	338.591 ± 0.375^d^	14,619.65 ± 74.396^d^	377.495 ± 0.347^d^	1,479.425 ± 61.871	13,517.72 ± 14.083^a^	14,997.15 ± 74.370^a^
K	X	1,643.087 ± 4.357^d^	15,129.18 ± 31.175^a^	24.811 ± 0.054^b^	327.686 ± 0.624^b^	16,772.36 ± 34.529^a^	352.498 ± 0.591^c^	1,667.899 ± 4.356^d^	15,456.96 ± 31.799^a^	17,124.86 ± 35.120^a^
	T	1,257.457 ± 1.936^b^	14,988.99 ± 8.0326^d^	36.758 ± 0.065^a^	333.375 ± 0.418^a^	16,246.44 ± 11.774^d^	370.133 ± 0.397^a^	1,294.182 ± 1.890^b^	15,322.36 ± 7.995^d^	16,616.58 ± 11.375^d^
	P	1,402.696 ± 53.039^c^	13,504.93 ± 19.1567^c^	38.881 ± 0.131^c^	334.179 ± 0.315^a^	14,907.62 ± 43.934^c^	373.061 ± 0.226^b^	1,441.577 ± 52.913^c^	13,839.11 ± 19.252^c^	15,280.68 ± 44.061^c^
	M	1,186.63 ± 3.597^a^	16,122.9 ± 16.065^b^	37.701 ± 0.043^d^	328.435 ± 0.315^b^	17,309.53 ± 23.273^b^	366.137 ± 0.272^d^	1,224.344 ± 3.544^a^	16,451.33 ± 16.377^b^	17,675.61 ± 23.573^b^
L	X	1,717.63 ± 0.276^a^	12,514.78 ± 29.7182^a^	39.224 ± 0.023^c^	318.224 ± 0.584^b^	14,232.41 ± 24.318^a^	357.448 ± 0.608^c^	1,756.866 ± 0.296^a^	12,833.01 ± 29.133^a^	14,589.86 ± 23.827^a^
	T	1,574.087 ± 38.737^b^	13,261.45 ± 17.506^b^	40.108 ± 0.175^d^	353.354 ± 0.240^a^	14,835.54 ± 52.955^b^	393.462 ± 0.242^a^	1,614.195 ± 38.833^b^	13,614.8 ± 17.480^b^	15,229 ± 53.176^b^
	P	1,487.087 ± 69.653^ab^	13,325.22 ± 22.808^c^	31.688 ± 0.059^a^	385.487 ± 0.854^b^	14,812.3 ± 71.373^b^	417.176 ± 0.811^b^	1,518.776 ± 69.594^ab^	13,710.7 ± 23.313^c^	15,229.48 ± 72.111^b^
	M	1,795.435 ± 209.201^ab^	13,952.46 ± 38.313^d^	33.099 ± 0.010^b^	352.327 ± 0.375^c^	15,747.9 ± 178.296^c^	385.427 ± 0.365^d^	1,828.535 ± 209.19^ab^	14,304.79 ± 38.498^d^	16,133.33 ± 178.358^c^

Note

Comparison of different parts of the same brand with the same index, marked a, b, c, and d with different letters, indicates significant difference (*p* < .05).

**Figure 4:**
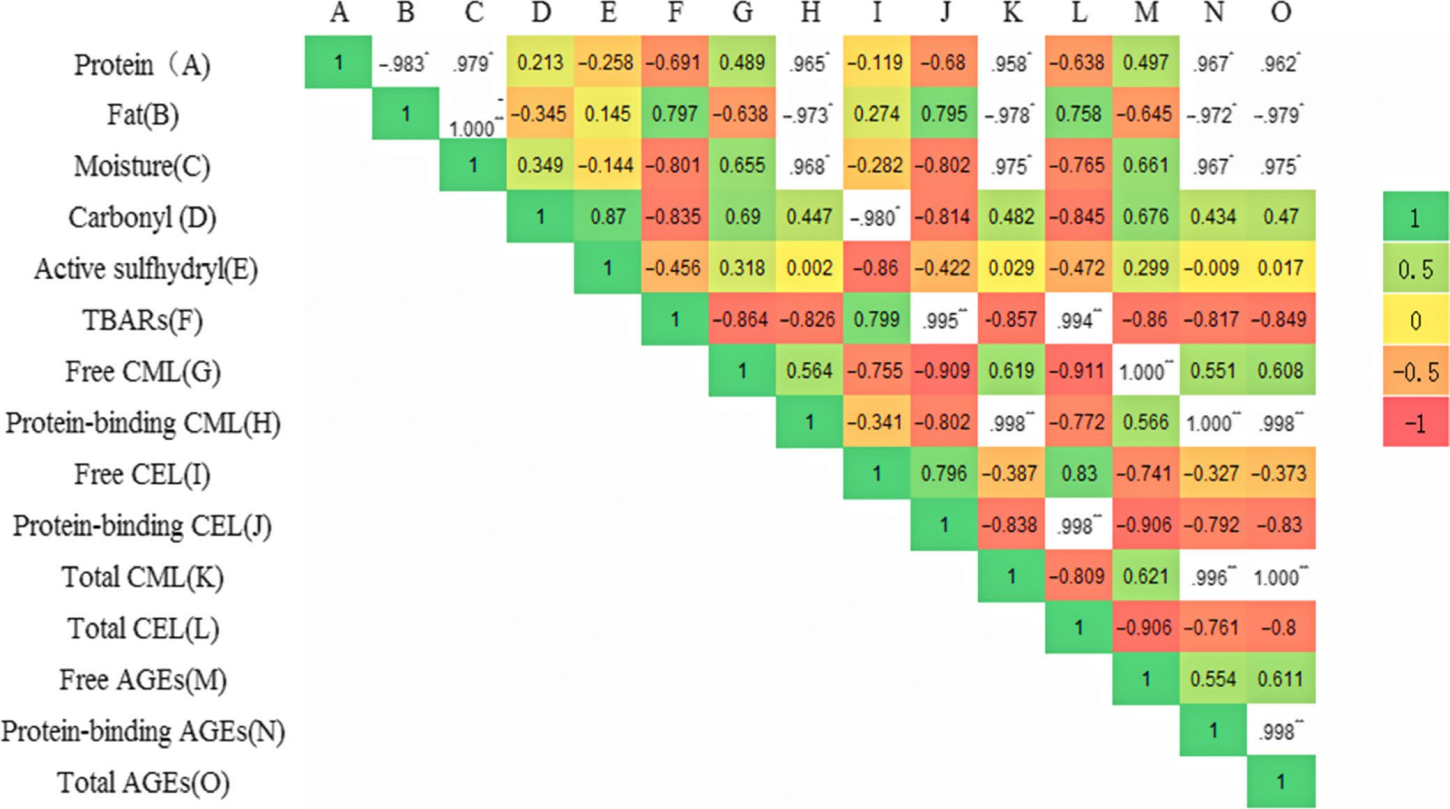
Correlation of protein, fat, moisture, carbonyl, active sulfhydryl, TBARs, free and protein‐binding forms of CML and CEL in breast (X), leg (T), skin (P), and the whole body (M) (four parts). * indicates that there is a significant difference, *p* < .05; ** indicates that there is a very significant difference, *p* < .01

Based on the analysis of the distribution of CML and CEL in X, T, P, and M, it could be concluded that the CML in free and protein‐binding forms was mainly distributed on X, and the CEL in free and protein‐binding forms was mainly distributed on P, and the content of protein‐binding state AGEs was much higher than that of free form in X, T, P, and M. This was also consistent with the results of the correlation in Figure 4.

### 3.3 Correlation

Correlation of protein, fat, moisture, carbonyl, active sulfhydryl, TBARs, free and protein‐binding forms of CML and CEL in four parts is shown in Figure 4. There was a significant positive correlation on fat content, protein‐binding CML, total CML, protein‐binding AGEs, and total AGEs (*p* < .05). However, there was a significant negative correlation on fat content, protein‐binding CML, total CML, protein‐binding AGEs, and total AGEs (*p* < .05). The correlation of moisture was consistent with that of protein.

Furthermore, according to the correlation analysis of Figure 4, protein and moisture mainly affected the formation of CML, especially protein‐binding CML, because meat protein contained a large amount of lysine, and that was the precursor of CML (Niu et al., 2018). In general, the moisture content was lower in sample, the Maillard reaction would be occurred stronger, and the more AGEs content would be formed, so there should be a negative correlation between the two (Lund & Ray, 2007), but our results showed a significant positive correlation, which indicated that different processing methods lead to the fluctuation of moisture content even in the same part of chicken. These fluctuations were affected by the Maillard reactions at different processing treatments that promoted the CML formation and accumulation at X, T, P, and M. The relationship between the fluctuation of moisture content and the key processing, such as frying, brine boiling, and secondary sterilization, needs to be further studied.

For oxidation, protein carbonyl showed a significant negative correlation with free form CEL (*p* < .05). The active sulfhydryl was also negatively correlated with free CEL with a correlation coefficient of −0.86 (*p* > .05). However, there was a significant positive correlation on TBARs, protein‐binding CEL, and total CEL (*p* < .01). Carbonyl content and free CEL had a significant negative correlation (*p* < .05), but a highly negative correlation with total CEL (*p* > .05). That means excessive protein oxidation during processing inhibited CEL formation. One reason was mainly that CEL was generated by CML; protein oxidation decreased the precursor substances formed by CML. For example, the unique flavor of processed meat products would be formed during the protein oxidation (Zhu, Lee, Mendonca, & Ahn, 2004). Another was that protein oxidation would lead to protein degradation, and moderate oxidation promotes CML to form some active sites, which led to the increase in levels of CEL. However, excessive oxidation destroyed the spatial structure of the protein, and the active site may be blocked by other groups; CEL has resulted in decrease (Shengmin et al., 2007).

The results of fat and protein oxidation were similar. Figure 4 shows that fat oxidation could significantly promote the protein‐binding form CEL and total CEL. This phenomenon exhibited that the fat oxidation may could produce a large amount of active oxygen free radicals. On the one hand, these free radicals were involved in the Maillard reaction that accelerated the formation of AGEs (Yu et al., 2016), and on the other hand, it could activate some sites such as protein and peptide ε‐NH_2_, which produced a large number of dicarbonyl compounds, such as GO and MGO accelerating CML to form CEL (Ahmed, Thorpe, & Baynes, 1986; Shibamoto, 2006). The processing of braised chicken involved complicated frying, braising, and secondary sterilization. These techniques are the essence of traditional Chinese meat processing. However, specific to the formation rules of free and protein‐binding forms of AGEs of braised chicken in each process, the degree of oxidation in this process was not clear whether to promote or inhibit AGEs in braised chicken.

## 4 CONCLUSION

In conclusion, the content and distribution of free and protein‐binding AGEs were different in different parts of braised chicken. Both CML and CEL existed in a large number of protein‐binding states. The protein‐rich parts such as X and T were dominated by CML, while the fat‐rich parts such as P were dominated by CEL. Protein oxidation promoted the formation of protein‐binding CML at different sites, while fat oxidation promoted the formation of CEL at different sites. However, the processing technology of braised chicken was so complicated that the formation and oxidation rules of AGEs need to be further studied. In a word, this paper provided some important data to show the CML and CEL contents in different parts of braised chicken and tried to analyze the relationship between the AGEs formation in different braised sites and the oxidation reaction.

## CONFLICT OF INTEREST

The authors declare that they have no conflict of interest.

## ETHICAL APPROVAL

The study did not involve any human or animal testing.

## INFORMED CONSENT

Written informed consent was obtained from all study participants.
